# Squamous and Respiratory Metaplasia After Olfactory Mucosal Resection

**DOI:** 10.3389/fnins.2021.695653

**Published:** 2021-07-20

**Authors:** Eri Mori, Rumi Ueha, Kenji Kondo, Shotaro Funada, Hajime Shimmura, Kai Kanemoto, Hirotaka Tanaka, Hironobu Nishijima, Nobuyoshi Otori, Tatsuya Yamasoba, Hiromi Kojima

**Affiliations:** ^1^Department of Otorhinolaryngology, The Jikei University School of Medicine, Tokyo, Japan; ^2^Swallowing Center, The University of Tokyo Hospital, Tokyo, Japan; ^3^Department of Otolaryngology and Head and Neck Surgery, Faculty of Medicine, University of Tokyo, Tokyo, Japan

**Keywords:** olfactory mucosa, olfactory receptor neurons, smell, olfactory impairment, olfactory ensheathing cells

## Abstract

Resection of the olfactory mucosa (OM) is sometimes unavoidable during surgery; however, it is not known whether the OM can completely recover thereafter. The aim of this study was to uncover whether the OM fully recovers after mucosal resection and describe the process of OM regeneration. 8-week-old male Sprague–Dawley rats (*n* = 18) were subjected to OM resection at the nasal septum; six rats were euthanized for histological examination 0, 30, and 90 days after surgery. Immunohistochemistry was performed to identify olfactory receptor neuron (ORN) lineage cells [mature and immature ORNs and ORN progenitors, and olfactory ensheathing cells (OECs)], as well as dividing and apoptotic cells. Squamous and respiratory metaplasia and inflammatory cell infiltration were also assessed. On day 30 after resection, the mucosa had regenerated, and mainly contained thin nerve bundles, basal cells, and immature ORNs, with a few mature ORNs and OECs. On day 90, the repaired nasal mucosa had degenerated into stratified squamous or ciliated pseudostratified columnar epithelia, with reducing ORNs. The lamina propria contained numerous macrophages. Partial regeneration was observed within 1 month after OM resection, whereas subsequent degeneration into squamous and respiratory epithelia occurred within 3 months. Given the poor persistence of ORNs and OECs, OM resection is likely to result in olfactory impairment. Overall, surgeons should be cautious not to injure the OM during surgery.

## Introduction

Degeneration and subsequent regeneration of the olfactory epithelium (OE) have been reported to be associated with various inciting causes including drug abuse (Sakamoto et al., 2007), cigarette smoking (Kim et al., 2011; Ueha et al., 2016a), and chemical exposure (Cancalon, 1982; Ueha et al., 2016b); these studies have demonstrated that the OE can regenerate as long as the basal cells are preserved. It is known that in severe injury models, depleting basal cells impairs OE regeneration and induces OE degeneration with time (Child et al., 2018). However, our knowledge regarding the recovery or regeneration of the OE after mechanical injury of all layers containing the basal cells, the Bowman’s glands of the lamina propria (LP), the olfactory nerve bundles, and olfactory ensheathing cells (OECs; Doucette, 1990), is currently limited.

Endoscopic sinus surgery is employed as a standard treatment for nasal sinus diseases such as chronic rhinosinusitis and nasal tumors and as a surgical approach to reach the skull base. It is well-known that complications and olfactory disruption occur after nasal surgery (Pfaar et al., 2004; Alobid et al., 2013). The removal of lesions in the olfactory cleft (OC) aims to open the pathway to the olfactory mucosa (OM), thereby maximizing olfactory functional outcomes (Jiang and Liang, 2020). However, this removal of lesions in the OC (Hurtt et al., 1988; Delank and Stoll, 1998) or the approach of OM removal remains controversial because of the risk of iatrogenic hyposmia or anosmia secondary to OM damage. Thus, there are no reasonable surgical approaches for the OC that preserve the sense of smell. To develop better surgical options, it is necessary to confirm whether the OM can fully recover after surgical disruption.

The aim of the present study was to address these questions. We successfully used a rat model to investigate the process of OM regeneration after mucosal resection and determine whether the OM recovers to its original state.

## Materials and Methods

### Animals

Eight-week-old male Sprague–Dawley rats weighing 330–360 g were purchased from CLEA Japan, Inc. (Tokyo, Japan) and housed in a temperature-controlled environment under a 12-/12-h light/dark cycle, with access to food and water *ad libitum*. All animal experiments were conducted in accordance with institutional guidelines and with the approval of the Animal Care and Use Committee of the University of Tokyo (Approval No. P19-057).

### Experimental Protocols and Surgical Procedure

**FIGURE 1:**
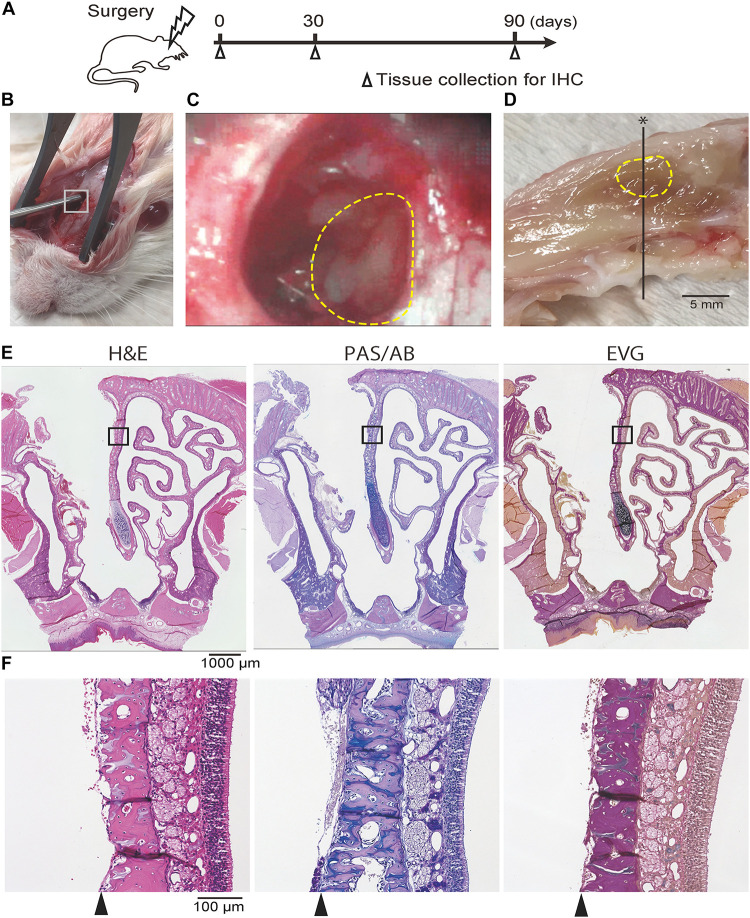
**(A)** Experimental timeline. **(B, C)** Photographic images of the surgical procedure. The box in **(B)** indicates the area where the hole was drilled; this is shown at a higher magnification in **(C)**. The dotted line indicates the area where the nasal mucosa was resected. **(D)** Mid-sagittal section of the rat nose. The dotted line indicates the area where the nasal mucosa was resected. The black line represents the line along which sections were cut. **(E,F)** Representative images of sections of the rat nose taken after partial nasal mucosal resection on day 0; hematoxylin and eosin (H&E) staining, Periodic Acid-Schiff and Alcian Blue (PAS/AB) staining, and Elastica van Gieson (EVG) staining are shown. The insets in **(E)** are shown at higher magnification in (**F**; **E**, 40× magnification; **F**, 200× magnification). The nasal mucosa, including the olfactory epithelium and subepithelial tissue, was completely resected along the perichondrium of the nasal septum (arrowheads).

Eighteen rats were used in this study. All rats were subjected to surgery, and six rats were euthanized for the collection of tissue samples on each of 0, 30, and 90 days after surgery (Figure 1A). The rats in the day 0 group were euthanized immediately after surgery.

Surgery was performed with the rats under general anesthesia induced by an intraperitoneal injection of ketamine hydrochloride (50 mg/kg) and xylazine hydrochloride (5 mg/kg). A vertical incision was made at the center of the dorsal aspect of the nasal bone. Following exposure of the right side of the frontal bone and the right nasal bone, a 4 mm × 6 mm hole was created by drilling from the infraorbital edge of the nasal bone and exposing the nasal septum (NS). To ensure reproducibility of the surgical technique, the surgery was always performed under a microscope by the same two researchers. The OM of the NS was partially resected using a curette and the septal bone was exposed (Figures 1B,C). The subcutaneous tissues were sutured to cover the hole in the facial bones, and the skin incision was then closed. The rats were allowed to recover in an approved animal care facility.

### Tissue Preparation

Nasal tissue specimens were harvested on days 0, 30, and 90 after surgery for histological analysis. Immediately after euthanizing the rats, the nasal cavity was gently irrigated with 10% formaldehyde to minimize mechanical damage to the OE. After harvest, the tissue samples were decalcified, dehydrated in graded ethanol solutions, and then embedded in paraffin.

### Histological Analyses

For histological analysis of the nasal mucosa, coronal sections were obtained from all samples at the level of the second palatal ridge of the hard palate (Uraih and Maronpot, 1990; Figure 1D). Paraffin sections of 4 μm thickness were deparaffinized in xylene and rehydrated in ethanol before staining. Hematoxylin and eosin staining was performed to evaluate the overall tissue structure, Elastica van Gieson (EVG) staining was used for connective tissue, periodic acid-Schiff and Alcian blue (PAS/AB) staining was used for goblet cells, and immunostaining was used to identify specific markers (Figures 1E,F). For immunostaining, deparaffinized sections were treated with 3% hydrogen peroxide to block endogenous peroxidase activity, and then incubated in Blocking One solution (Nacalai Tesque, Kyoto, Japan) to block non-specific immunoglobulin binding. After antigen retrieval, the samples were incubated with primary antibodies, followed by peroxidase-conjugated secondary antibodies; diaminobenzidine substrate was used as a chromogen.

The primary antibodies used in this study are listed in Table 1. The following antibodies were used to evaluate neurogenesis: sex-determining region Y-box 2 (SOX2), expressed by proliferating stem cells or progenitor cells in the basal layer of the OE; growth-associated protein 43 (GAP43), expressed by immature ORNs in the OE; olfactory marker protein (OMP), expressed by mature olfactory receptor neurons (ORNs) in the OE; cytokeratin 5 (CK5), expressed by quiescent horizontal basal cells in the OE and by squamous cells; p75 nerve growth factor receptor (p75), expressed by OECs in the OM (Wewetzer et al., 2005); Ki67, a cellular marker for proliferation; and caspase 3 (Cas3), a frequently activated cell death protease (Ueha et al., 2018). To assess inflammatory cell infiltration, CD3 and CD68 were used to detect T cells and macrophages, respectively, (Ueha et al., 2020). β4 tubulin (β4T) was used to detect ciliated respiratory cells (Jumat et al., 2015). The evaluation of mucosal regeneration was restricted to two areas in the two coronal sections at 500-μm intervals: the NS and dorsolateral (DL) area. Images were captured using a digital microscope camera (Keyence BZ-X700) with the 4× and 20× objective lenses. OMP^+^ ORNs, SOX2^+^ ORN progenitors, GAP43^+^ immature ORNs, CD3^+^ cells, CD8^+^ cells, Ki67^+^ dividing cells, and Cas3^+^ apoptotic cells in a 500-μm region of each area were counted in both treated and untreated sides. Ki67^+^ dividing cells and Cas3^+^ apoptotic cells in the NS were first counted in a 1,000-μm OE range, and then, to unify the results from other cells, the cell number was recalculated for the 500-μm range. The number of each cell type was quantitatively analyzed using sections by single immunostaining for each antigen and counterstaining with hematoxylin.

**TABLE 1:** Information regarding the primary antibodies used in this study.

**Primary antibody**	**Sourse**	**Catalog No.**	**Host**	**Type**	**Dilution**
SOX2	Abcam (Cambridge, United Kingdom),	ab92494	Rabbit	Monoclinal	1:300
GAP43	Novus Biologicals (Centennial, CO, United States)	NB300-143B	Rabbit	Polyclonal	1:1000
OMP	Wako (Tokyo, Japan)	019-22291	Goat	Polyclonal	1:8000
CK5	Abcam (Cambridge, United Kingdom),	ab52635	Rabbit	Monoclinal	1:200
p75	Abcam (Cambridge, United Kingdom),	ab52987	Rabbit	Monoclinal	1:300
Ki67	Novus Biologicals (Centennial, CO, United States)	NB600-1252	Rabbit	Monoclinal	1:200
Cleaved capase-3	Cell Signaling (Danvers, MA, United States)	9661	Rabbit	Polyclonal	1:300
β4 tublin	MilliporeSigma (Darmstadt, Germany)	T7941	Mouse	Monoclinal	1:300
CD3	Nichirei (Tokyo, Japan)	413601	Rabbit	Monoclinal	1:300
CD68	Abcam (Cambridge, United Kingdom),	ab31630	Mouse	Monoclinal	1:200

Statistical analyses were performed; specifically, a two-way analysis of variance test with Sidak’s *post-hoc* tests for comparisons among multiple groups was performed using GraphPad Prism software (version 6.7; GraphPad Software, Inc., San Diego, CA, United States, www.graphpad.com). Results with *P* < 0.05 were considered significant.

## Results

### Mucosa of the NS Showed Signs of Regeneration 30 Days After Mucosal Resection, but Degenerated Within 3 Months

**FIGURE 2:**
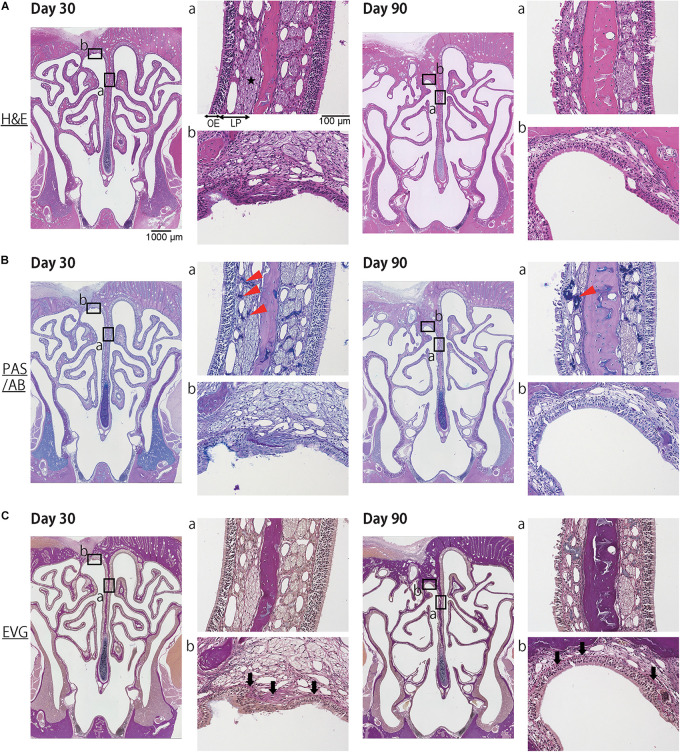
Representative images of sections of the nasal mucosa taken 30 and 90 days after mucosal resection (lower magnification, 40×; higher magnification, 200×). Hematoxylin and eosin (H&E) staining, Periodic Acid-Schiff and Alcian Blue (PAS/AB) staining, and Elastica van Gieson (EVG) staining are shown. **(A)** H&E staining. On day 30, the mucosa of the nasal septum (NS: a), including the olfactory epithelium (OE) and lamina propria (LP), had regenerated to some extent. The repaired OE consisted of stratified ciliated columnar cells, and the LP consisted of olfactory nerve bundles (star), connective tissue, and vessels. In the dorsolateral area (DL: b), the drilled hole had closed with the development of a new bone and connective tissue. On day 90, the OE in the NS had thinned, and its appearance had changed. The area occupied by olfactory nerve bundles was diminished. In the DL, the new osseous tissue had proliferated excessively and become covered by ciliated pseudostratified columnar epithelium. **(B)** PAS/AB staining revealed the presence of Bowman’s glands in the LP (red arrowheads). **(C)** EVG staining was used to highlight connective tissue, such as collagen fibers (stained red: black arrows).

Histological examination revealed that the mucosa of the NS, including the epithelium and the subepithelial tissue of the LP, had been repaired and begun to regenerate by day 30 after mucosal resection. However, the regenerated tissue was thin in comparison to the nasal mucosa of the un-operated side (Figure 2A). PAS/AB staining showed the regeneration of olfactory nerve bundles and Bowman’s glands in the LP (Figure 2B). EVG staining revealed that the structure of the mucosa, including the epithelium, basement membrane, and LP, was preserved in the reconstructed tissue (Figure 2C). In the DL area, the drilled hole was closed by the development of a new bone and connective tissue. The regenerated epithelium in the DL area was thin, and the basement membrane had a disordered appearance (Figure 2A). However, on day 90 after mucosal resection, images of the DL area showed excessive growth of new osseous tissue, whereas images of the NS revealed thinning of the nasal mucosa, including that of the stratified epithelium and LP. Furthermore, the area occupied by olfactory nerve bundles and Bowman’s glands had decreased (Figures 2A,B).

### Mature ORNs Did Not Regenerate After Mucosal Resection, Although Immature Neurons Were Initially Observed

**FIGURE 3:**
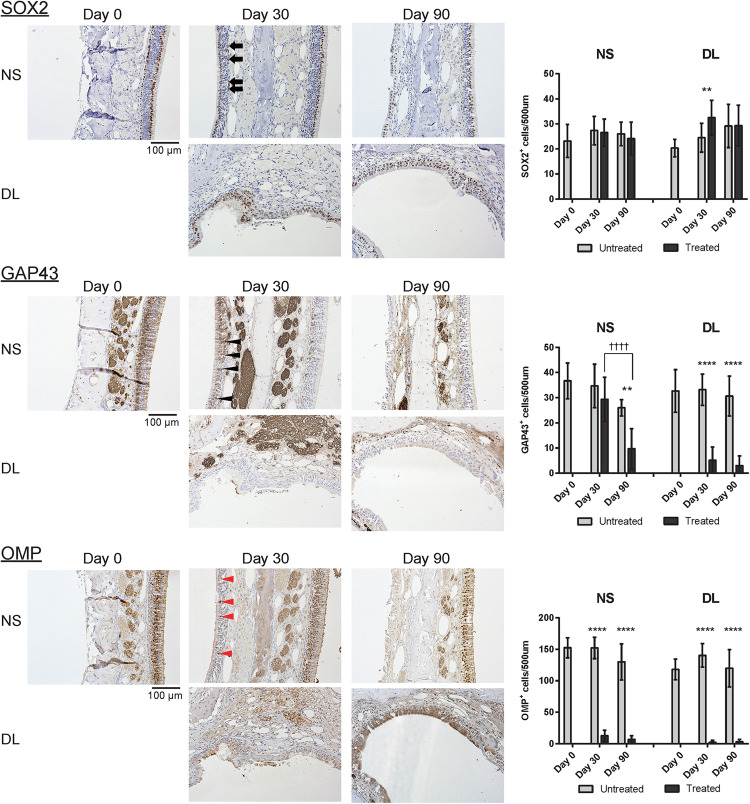
Representative images of sections of the nasal mucosa stained with antibodies against OMP, SOX2, and GAP43 (magnification, 200×). OMP^+^ mature olfactory receptor neurons (ORNs), SOX2^+^ ORN progenitors, and GAP43^+^ immature ORNs in the epithelium were identified by immunohistochemical staining (brown). On day 30, mature OMP^+^ ORNs were sparse (red arrowheads), whereas GAP43^+^ immature ORNs (black arrowheads) were more frequently observed in the epithelium of the nasal septum (NS). In the upper lateral (DL) area, SOX2^+^ cells (black arrows) but no GAP43^+^ or OMP^+^ cells were present in the epithelium. On day 90, neither GAP43^+^ immature ORNs nor OMP^+^ mature ORNs were present in the epithelium of the NS or DL area. Graphs show the number of each cell type per 500 μm of basal layer length. Asterisks indicate a significant difference between the treated and untreated groups. Daggers indicate a significant difference between time points. **P < 0.01; ****P < 0.0001, ^††††^*P* < 0.0001 (*n* = 6, two-way analysis of variance).

**FIGURE 4:**
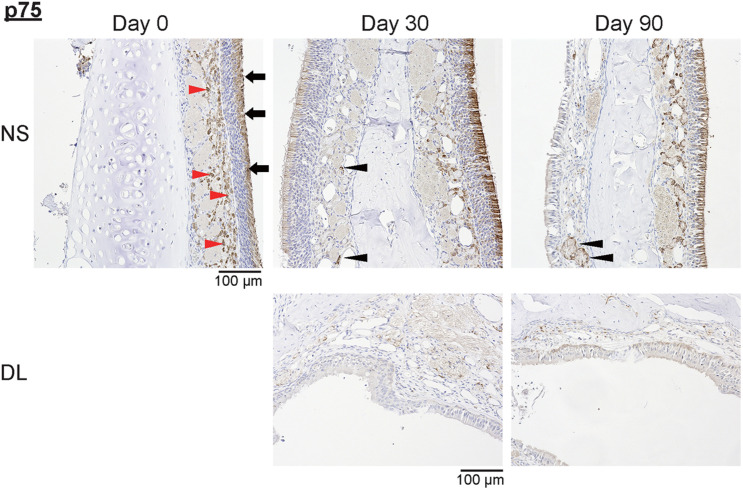
Representative images of sections of the nasal mucosa stained with antibodies against p75 (magnification, 200×). NS, nasal septum; DL, dorsolateral area. P75^+^ olfactory ensheathing cells (red arrowheads) and p75^+^ uppermost superficial layer cells (black arrowheads) in the nasal mucosa were identified by immunohistochemical staining (brown).

Next, to investigate whether the repaired nasal mucosa displayed characteristics typical of OE, we examined the expression of SOX2, GAP43, and OMP in the regenerated epithelium. The analysis of the OE of the NS 30 days after mucosal resection showed that SOX2^+^ ORN progenitors were present in the basal layer, whereas GAP43^+^ immature ORNs were detected above the basal layer, although OMP^+^ mature ORNs were scarce (Figure 3). On day 30, in the OE of the DL area, only SOX2^+^ ORN progenitors were detected. On day 90 after mucosal resection, the epithelium of the NS and DL area contained SOX2^+^ cells, but neither GAP43^+^ immature ORNs nor OMP^+^ mature ORNs could be almost recognized (Figure 3), suggesting that the OE that regenerate after mucosal resection might degenerate within 3 months, and show properties that are characteristic of other epithelial types. On days 30 and 90, weakly OMP-positive and strongly GAP43-positive nerve fibers could be observed in the LP on the treated side. However, OMP staining on the treated side was weaker than that on the untreated side. p75^+^ OECs were widely present in the LP in the OM, and p75 was also expressed in the uppermost superficial layer of the OE (Figure 4, day 0, the untreated side). On day 30, after mucosal resection, a few OECs were present in the LP, but they had not fully regenerated by day 90. In the untreated side, the number of OECs appeared to be decreasing on day 30, but by day 90, the distribution of OECs in the LP of the OM had recovered to almost the same level as on day 0 (Figure 4).

### Repaired Nasal Mucosa Degenerated Into Stratified Squamous or Ciliated Pseudostratified Columnar Epithelia 90 Days After Mucosal Resection

**FIGURE 5:**
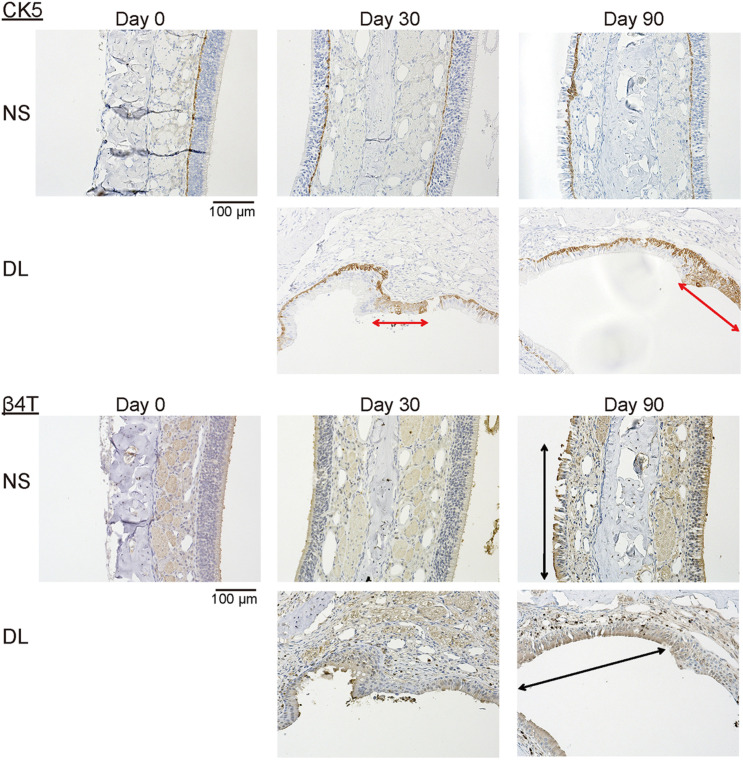
Representative images of sections of the nasal mucosa stained with antibodies against CK5 and β4T (magnification, 200×). CK5 expression was recognized in a part of the repaired nasal epithelium in the upper lateral (DL) area on days 30 and 90 after surgery (red arrows). β4T was expressed in a part of the villous brush border of the columnar epithelium of the nasal septum (NS) and the DL area on day 90 (black arrows).

**FIGURE 6:**
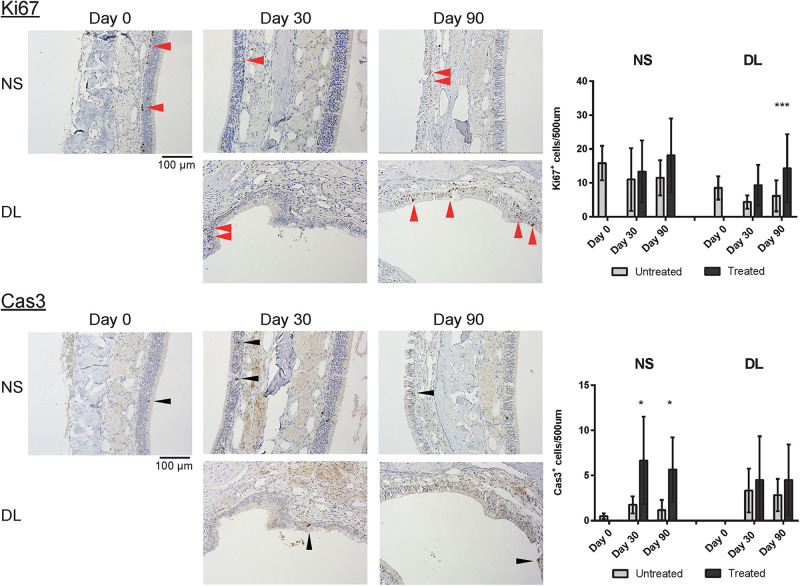
Representative images of sections of the nasal mucosa stained with antibodies against Ki67 and Cas3 (magnification, 200×). Ki67^+^ dividing cells (red arrowheads) and Cas3^+^ apoptotic cells (black arrowheads) were rarely present in the nasal epithelium on day 30, whereas several Ki67^+^ cells were observed in the DL area on day 90. Asterisks indicate a significant difference between the treated and untreated groups. *P < 0.05; ***P < 0.001 (*n* = 6, two-way analysis of variance).

To determine the properties of the repaired epithelium in terms of the presence of squamous or respiratory metaplasia and the levels of cell division and apoptosis, we examined the expression of CK5, β4T, Ki67, and Cas3 in the nasal epithelium. Weak CK5 staining was observed in the DL area even on day 30 after mucosal resection, but this staining strengthened by day 90; however, CK5 expression was not detected in the epithelium of the NS, except in the basal cells (Figure 5). Although no definite expression of β4T was detected in either the NS or DL areas on day 30, β4T expression was observed in a small part of the DL area and most parts of the NS on day 90 (Figure 5). Ki67^+^ dividing cells were rarely present in the nasal epithelium of the NS area on days 30 and 90, whereas several Ki67^+^ cells were observed in the DL area on day 90. Some Cas3^+^ apoptotic cells were present in the nasal epithelium of the NS area on days 30 and 90, whereas Cas3^+^ cells were observed in the DL area on days 30 and 90 (Figure 6). Given the positive staining reactions for CK5 and β4T in the DL area on day 90 after mucosal resection, it could be inferred that the nasal epithelium partially degenerated into stratified squamous and ciliated pseudostratified columnar epithelia.

### CD68^+^ Macrophages Were Abundant in the Mucosa of the NS and Upper Lateral Area, Especially in the Subepithelial Tissue, on Day 90 After Mucosal Resection

**FIGURE 7:**
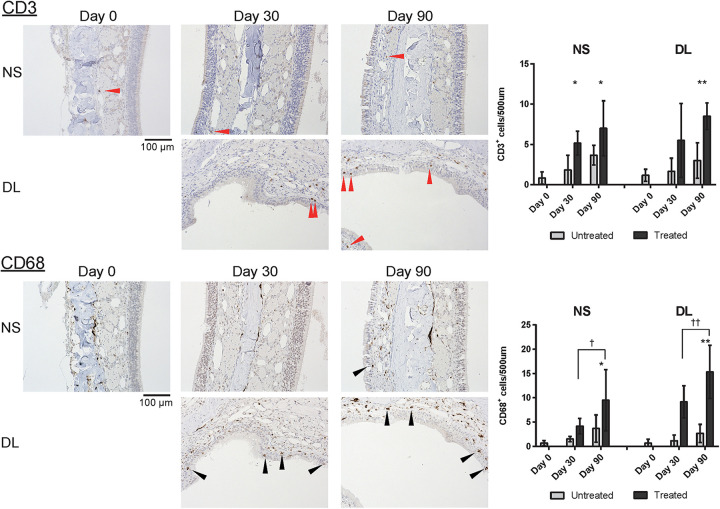
Representative images of sections of the nasal mucosa stained with antibodies against CD3 and CD68 (magnification, 200×). NS, nasal septum; DL, dorsolateral area. CD3^+^ T lymphocytes (red arrowheads) and CD68^+^ macrophages (black arrowheads) in the nasal mucosa were identified by immunohistochemical staining (brown). Graphs show the number of each cell type per 500 μm of basal layer length. *P < 0.05; **P < 0.01; ^†^*P* < 0.05; and ^‡^*P* < 0.01 (*n* = 6, one-way analysis of variance).

Finally, to investigate inflammatory cell infiltration in the nasal mucosa, we assessed the presence of CD3^+^ T cells and CD68^+^ macrophages. An increase in the number of CD3^+^ cells was observed in the mucosa of the NS and DL area on days 30 and 90; especially, the number of CD3^+^ cells was significantly increased in the DL area on day 90 (Figure 7). CD68^+^ cells were more abundant in the mucosa of the NS and DL area on day 90. This suggests that CD68^+^ macrophages induce the degeneration of connective tissue and the nasal epithelium (Figure 7).

Overall, although there was some evidence of OM regeneration 30 days after resection, ORNs did not persist in the regenerating areas, and metaplasia into squamous and respiratory epithelium occurred within 90 days of surgery.

## Discussion

In this study, we investigated the patterns of mucosal repair following OM resection. It is well-known that OE can regenerate after surgical interventions, such as olfactory bulb ablation (Booth et al., 1981) or transection of the olfactory nerves (Costanzo, 1985). In the rat model used in this study, the full thickness of the OM was removed, including the LP, basal cells, olfactory nerve bundles, and OECs, and the bones of NS were exposed. Our results demonstrated that although full-thickness excision of the OM was followed by regeneration of the nasal mucosa in the short term, it led to degeneration over time.

Although some mature ORNs and OECs were present, the basal cells, immature ORNs, Bowman’s glands, and nerve bundles in the LP regenerated after 30 days, and the OM appeared to be regenerating normally, albeit at a relatively slow pace. At 90 days after OM resection, the axons of OSN had regenerated to some extent; however, few cells with ORN or olfactory ensheathing characteristics were identified in the repaired OM, and the LP appeared to be predominantly composed of connective tissue. OMP and p75 were expressed weakly in the treated side compared with those in the untreated side. These results suggest that nerve axons can regenerate, whereas regeneration of the OECs could be insufficient. In addition, this degeneration may have been induced by inappropriate connections between the OE and olfactory nerve bundles or Bowman’s glands, and by the limited neuroregenerative capacity of olfactory stem cells after injury (Child et al., 2018), resulting in thinning of the repaired nasal mucosal epithelium. Considering that mature ORNs or OECs were not present in the repaired epithelium on day 30, the normal processes of olfactory adaptation and perception could not occur properly, even if odorants reached the cilia of the epithelial cells. This decrease in odor transduction may have induced the structural changes in the repaired nasal mucosa that were observed between 30 and 90 days after OM resection (Kikuta et al., 2015).

Submucosal chronic inflammation can also affect OE degeneration (Chen et al., 2019). As the number of macrophages in the LP was higher on day 90 than on day 30, it could be suggested that macrophages were involved in the changes in the biophysical properties of the repaired epithelium. The limited number of dividing and apoptotic cells suggests that functional insufficiency, rather than apoptosis, contributes to the observed structural changes in the repaired mucosa. Similarly, the process of regeneration after injury of respiratory mucosa has been extensively studied (Moriyama et al., 1996; Shaw et al., 2001; Choi et al., 2017). Respiratory mucosa regenerates when the basal membrane is intact after injury. However, when the basal membrane is damaged, mucosa forms from squamous epithelium (Cöloðlu et al., 2006). Additionally, the respiratory mucosa cannot regenerate following complete removal; instead, it is substituted by dense connective tissue (Benninger et al., 1989) and covered with scattered cilia cells (Moriyama et al., 1996). To retain the OM, it may be important to only remove the surface of the OE and not expose the surface of the bone.

The nature of epithelial degeneration that occurred by day 90 was different in the NS and DL areas. The epithelium of the NS was more likely to degenerate into ciliated pseudostratified columnar epithelium; interestingly, this is similar to the degeneration observed with aging (Naessen, 1971; Doty and Kamath, 2014). In the DL area, after the removal of all tissue including bone, the regenerated tissue had different characteristics, and had degenerated into a combination of stratified squamous and respiratory epithelia. This difference in degeneration may be explained by the presence of the septal nasal cartilage in the NS area as a support base, acting to prevent mucosal degeneration after mucosal resection (Ozdemir et al., 2019). Provided that mucosal regeneration advances smoothly following OM resection, olfactory functional outcomes can be maximized after surgery in the OC, allowing the range of surgical options to be broadened. From our results, interventions to interrupt the metaplasia of epithelial cells with ORNs, potentially into respiratory or squamous populations, might be effective in preventing the loss of OE and olfactory function, and should be performed between days 30 and 90 after mucosal resection. Moreover, interventions to promote appropriate differentiation of immature ORNs to mature ORNs must be performed within 30 days.

As with any animal study, a limitation of our results is that we cannot be sure that the processes identified in this rat model are equivalent to those in humans; rodents have vomeronasal sensory neurons in the nasal cavity, while humans do not. In addition, the evaluation was only performed 30 and 90 days after surgery and, considering that the process of regeneration could be influenced by various time-dependent factors, including inflammation, additional data from other time points would have been desirable. To discover possible effective interventions required to avoid degeneration, it is necessary to further investigate molecular changes.

In conclusion, this study showed that the OE can regenerate to some extent, as cells with the ORN lineage appear in regenerating areas within 30 days of OM resection. It is likely that olfaction would be affected by OM resection, considering the persistence of immature and mature ORNs in the regenerated epithelium. Thus, surgeons should be cautious not to completely injure the OM. However, given the long-term structural changes observed in this study, interventions to promote the appropriate differentiation of ORNs and interrupt squamous and respiratory metaplasia may support mucosal regeneration following OM resection.

## Data Availability Statement

The raw data supporting the conclusions of this article will be made available by the authors, without undue reservation.

## Ethics Statement

The animal study was reviewed and approved by Animal Care and Use Committee of the University of Tokyo (Approval No. P19-057).

## Author Contributions

EM and RU carried out all the processes of this study, including study conceptualization and design, data acquisition, data analysis, and data interpretation. SF, HS, KaK, and HT contributed to data acquisition. HN, KeK, NO, TY, and HK conceived the project. All authors contributed to the article and approved the submitted version.

## Conflict of Interest

The authors declare that the research was conducted in the absence of any commercial or financial relationships that could be construed as a potential conflict of interest.
